# Meta‐analysis of genome‐wide DNA methylation and integrative omics of age in human skeletal muscle

**DOI:** 10.1002/jcsm.12741

**Published:** 2021-06-30

**Authors:** Sarah Voisin, Macsue Jacques, Shanie Landen, Nicholas R. Harvey, Larisa M. Haupt, Lyn R. Griffiths, Sofiya Gancheva, Meriem Ouni, Markus Jähnert, Kevin J. Ashton, Vernon G. Coffey, Jamie‐Lee M. Thompson, Thomas M. Doering, Anne Gabory, Claudine Junien, Robert Caiazzo, Hélène Verkindt, Violetta Raverdy, François Pattou, Philippe Froguel, Jeffrey M. Craig, Sara Blocquiaux, Martine Thomis, Adam P. Sharples, Annette Schürmann, Michael Roden, Steve Horvath, Nir Eynon

**Affiliations:** ^1^ Institute for Health and Sport (iHeS) Victoria University, Footscray Melbourne Vic. Australia; ^2^ Faculty of Health Sciences & Medicine Bond University Gold Coast Qld Australia; ^3^ Centre for Genomics and Personalised Health, Genomics Research Centre, School of Biomedical Sciences, Institute of Health and Biomedical Innovation Queensland University of Technology (QUT) Kelvin Grove Qld Australia; ^4^ German Center for Diabetes Research (DZD) München‐Neuherberg Germany; ^5^ Division of Endocrinology and Diabetology, Medical Faculty Heinrich Heine University Düsseldorf Germany; ^6^ Department of Experimental Diabetology German Institute of Human Nutrition Potsdam‐Rehbruecke (DIfE) Nuthetal Germany; ^7^ School of Health, Medical and Applied Sciences Central Queensland University Rockhampton Qld Australia; ^8^ Université Paris‐Saclay, UVSQ, INRAE, BREED Jouy‐en‐Josas France; ^9^ Ecole Nationale Vétérinaire d'Alfort, BREED Maisons‐Alfort France; ^10^ Univ Lille, Inserm, CHU Lille, Pasteur Institute Lille, U1190 Translational Research for Diabetes, European Genomic Institute of Diabetes Lille France; ^11^ Department of Metabolism, Digestion and Reproduction Imperial College London London UK; ^12^ IMPACT Institute Deakin University, Geelong Waurn Ponds Campus Geelong Vic. Australia; ^13^ Epigenetics, Murdoch Children's Research Institute Royal Children's Hospital Parkville Vic. Australia; ^14^ Physical Activity, Sport & Health Research Group, Department of Movement Sciences KU Leuven Leuven Belgium; ^15^ Institute for Physical Performance Norwegian School of Sport Sciences Oslo Norway; ^16^ Institute for Clinical Diabetology, German Diabetes Center, Leibniz Center for Diabetes Research Heinrich Heine University Düsseldorf Germany; ^17^ Department of Human Genetics and Biostatistics, David Geffen School of Medicine University of California Los Angeles Los Angeles CA USA

**Keywords:** Skeletal muscle, Ageing, Epigenetics, DNA methylation, Epigenetic clock, Meta‐analysis, Omics

## Abstract

**Background:**

Knowledge of age‐related DNA methylation changes in skeletal muscle is limited, yet this tissue is severely affected by ageing in humans.

**Methods:**

We conducted a large‐scale epigenome‐wide association study meta‐analysis of age in human skeletal muscle from 10 studies (total *n* = 908 muscle methylomes from men and women aged 18–89 years old). We explored the genomic context of age‐related DNA methylation changes in chromatin states, CpG islands, and transcription factor binding sites and performed gene set enrichment analysis. We then integrated the DNA methylation data with known transcriptomic and proteomic age‐related changes in skeletal muscle. Finally, we updated our recently developed muscle epigenetic clock (https://bioconductor.org/packages/release/bioc/html/MEAT.html).

**Results:**

We identified 6710 differentially methylated regions at a stringent false discovery rate <0.005, spanning 6367 unique genes, many of which related to skeletal muscle structure and development. We found a strong increase in DNA methylation at Polycomb target genes and bivalent chromatin domains and a concomitant decrease in DNA methylation at enhancers. Most differentially methylated genes were not altered at the mRNA or protein level, but they were nonetheless strongly enriched for genes showing age‐related differential mRNA and protein expression. After adding a substantial number of samples from five datasets (+371), the updated version of the muscle clock (MEAT 2.0, total *n* = 1053 samples) performed similarly to the original version of the muscle clock (median of 4.4 vs. 4.6 years in age prediction error), suggesting that the original version of the muscle clock was very accurate.

**Conclusions:**

We provide here the most comprehensive picture of DNA methylation ageing in human skeletal muscle and reveal widespread alterations of genes involved in skeletal muscle structure, development, and differentiation. We have made our results available as an open‐access, user‐friendly, web‐based tool called *MetaMeth* (https://sarah‐voisin.shinyapps.io/MetaMeth/).

## Background

While human lifespan (i.e. the number of years alive) has increased by ~3.5 years per decade since 1900,^1^ healthspan (i.e. number of years spent in good health) has not increased to the same extent. In 2015, people lived 5 years longer than in 2000, but only 4.6 years longer in good health.^2^ Ageing leads to the progressive loss of muscle mass and strength, resulting in a disorder termed sarcopenia. Sarcopenia is a serious condition leading to an increased risk of many conditions including cancer, type 2 diabetes (T2D), and cardiovascular diseases.^3^ This process is driven by a host of adverse molecular changes in skeletal muscle with advancing age. Unravelling the molecular changes caused by ageing in skeletal muscle is the basic foundation for the development of drugs and targeted health‐related interventions to help prevent sarcopenia and maximize healthspan.

Epigenetics are modifications of DNA that confer on the cell the ability to remember a past event.^4^ Epigenetic changes are one of the *primary* hallmarks of ageing, leading to dysregulated nutrient sensing, mitochondrial dysfunction, and cellular senescence, which ultimately results in stem cell exhaustion and altered intercellular communication.^5^ The best characterized epigenetic modification in the context of ageing is DNA methylation. DNA methylation occurs at millions of CpG dinucleotides in the genome and changes considerably with age in various human tissues,^6^ including skeletal muscle.^7^, ^8^, ^9^ Age‐related DNA methylation changes in skeletal muscle may be one of the molecular mechanisms underlying sarcopenia, but the full picture is fragmentary. To date, four epigenome‐wide association studies (EWASs)^7^, ^8^, ^10^, ^11^ have probed age‐related DNA methylation changes in the muscle methylome, and all relied on relatively small sample sizes (*n* = 10–50). Studies relying on a small sample size fail to detect small effect sizes and can be prone to large error, so larger initiatives are needed to identify the comprehensive list of CpG loci that change in methylation with age in human skeletal muscle. Meta‐analyses significantly increase statistical power and are more likely to identify robust age‐related methylation sites.^12^ Current understanding of epigenetic ageing in skeletal muscle also remains incomplete as insight into the functional consequences of age‐related epigenetic changes remains poorly understood. Whether age‐related changes in DNA methylation in muscle cause or stem from changes in mRNA and protein expression is currently unknown.

To address these gaps, we performed a large‐scale bioinformatics analysis of DNA methylation, and mRNA and protein changes with age in human skeletal muscle. We integrated original DNA methylation data from our laboratory (the Gene SMART cohort) with available open‐access data from multiple repositories and published studies. Firstly, we aimed to identify robust age‐related CpGs in skeletal muscle in an EWAS meta‐analysis of age, combining *n* = 908 samples from 10 datasets. Second, we performed enrichment analyses to unravel the potential functional consequences of these robust age‐related DNA methylation changes. Thirdly, we integrated age‐related methylome changes with transcriptome and proteome changes in skeletal muscle using two external, large‐scale studies. Finally, we updated our skeletal muscle epigenetic clock^9^ with an additional 371 samples, reaching a total of 1053 human skeletal muscle methylomes from 16 datasets. Importantly, we have made the results of our analysis available as an open‐access, user‐friendly, interactive web‐based tool, *MetaMeth* (https://sarah‐voisin.shinyapps.io/MetaMeth/), enabling users to look at age‐related changes in any gene of interest across the muscle methylome, transcriptome, and proteome.

## Methods

### Epigenome‐wide association study meta‐analysis of age in skeletal muscle

We combined four datasets of genome‐wide DNA methylation in skeletal muscle [the Gene Skeletal Muscle Adaptive Response to Training (SMART),^13^ the Limb Immobilisation and Transcriptional/Epigenetic Responses (LITER) study,^9^ the Biological Atlas of Severe Obesity (ABOS) study,^14^ and the Epigenetica & Kracht (EPIK) study^10^], with five datasets from the open‐access Gene Expression Omnibus platform (GSE49908,^8^ GSE50498,^7^ GSE114763,^15^ GSE38291,^16^ and GSE135063^17^), and the Finland‐United States Investigation of NIDDM Genetics (FUSION) study^18^ (phs000867.v1.p1). These summed up to a total of *n* = 908 skeletal muscle samples collected from men and women across the lifespan (age range 18–89 years old, Supporting Information, *Figure* S1 and *Table* S1). Samples were 98% Caucasian and 71% male (*Table* S1). We excluded cohorts from our recently published paper^9^ with a narrow age range (age standard deviation <5 years) as age‐related differences in DNA methylation cannot be detected if age is invariant; we also excluded datasets with a limited number of samples (*n* < 20) for robustness. Samples from the Gene SMART cohort (*n* = 234) include two batches, and our recently published paper^9^ only includes the first batch of 75 samples available on the Gene Expression Omnibus platform (GSE151407). The additional 159 samples from the second batch include both men and women, before and after exercise intervention.

Different preprocessing pipelines may result in DNA methylation differences between studies. To overcome this issue, we downloaded and preprocessed the data using the same pipeline for 9/10 datasets whose raw data were available (*Table* S1). Details on the preprocessing steps can be found elsewhere.^9^ We have also filtered out additional probes that have been identified as cross‐hybridizing by Pidsley *et al*.^19^ We did not preprocess all datasets together because age distributions varied widely between datasets (*Figure* S1). As age was confounded with dataset, normalizing datasets together may overcorrect/under‐correct DNA methylation profiles and artificially introduced noise. Therefore, we analysed each dataset separately and only then perform a meta‐analysis, which preserves each dataset's specificity while combining results across datasets. We conducted independent EWAS of age in skeletal muscle in each dataset, using linear models and moderated Bayesian statistics as implemented in *limma* ^20^; to isolate the contribution of age to DNA methylation variability, we regressed DNA methylation level against age and adjusted, when the dataset included these covariates, for sex, body mass index (BMI), diabetes status, batch, and time point (baseline/post‐intervention or training); we also added, when the dataset included repeated measures on the same individuals or related individuals, a random intercept using the duplicateCorrelation function to account for repeated measures from the same individuals or to account for twinship. We adjusted each EWAS for bias and inflation using the empirical null distribution as implemented in *bacon* (*Figure* S2).^21^ Inflation and bias in EWAS are caused by unmeasured technical and biological confounding, such as population substructure, batch effects, and cellular heterogeneity.^22^ The inflation factor is higher when the expected number of true associations is high (as it is for age); it is also greater for studies with higher statistical power.^21^ The figures we found (*Figure* S2) were consistent with the inflation factors and biases reported in an EWAS of age in blood.^21^

Results from the independent EWAS were combined using an inverse variance weighted meta‐analysis with METAL.^12^ We used METAL because it does not require all DNA methylation datasets to include every CpG site on the HumanMethylation arrays. Different sets of CpGs may be filtered out during preprocessing of each individual dataset, which means the overlap between the datasets is imperfect and a given CpG may only be present in five out of 10 datasets or eight out of 10 datasets. For robustness, we only included CpGs present in at least six of the 10 cohorts (649 250 CpGs). We used a fixed effects (as opposed to random effects) meta‐analysis, assuming one true effect size of age on DNA methylation, which is shared by all the included studies. Nevertheless, Cochran's *Q*‐test for heterogeneity was performed to test whether effect sizes were homogeneous between studies [a heterogeneity index (*I* ^2^) >50% reflects heterogeneity between studies]. The CpGs associated with age at a stringent meta‐analysis false discovery rate (FDR) <0.005 were considered differentially methylated positions (DMPs). We then identified differentially methylated regions (DMRs) (i.e. clusters of DMPs with consistent DNA methylation change with age) using the *dmrcate* package, at a Fisher's multiple comparison statistic <0.005, a Stouffer score <0.005, and a harmonic mean of the individual component FDRs <0.005.^23^ *dmrcate* works by smoothing the test statistic of CpGs separated by a maximum of 1000 bp using a Gaussian kernel; then, it models the smoothed test statistics, computes and corrects *P*‐values, and finally aggregates adjacent CpGs that are significant and within 1000 bp of each other. We focused on the DMRs for all downstream analyses, as DMRs remove spatial redundancy (CpG sites within ~500 bp are typically highly correlated^24^), and they may provide more robust and functionally important information than DMPs.^25^, ^26^

### Enrichment of differentially methylated regions in functional regions of the genome

We used a *χ* ^2^ test to compare the distribution of hypermethylated and hypomethylated DMRs with that of non‐DMRs (i) at different positions with respect to CpG islands, (ii) in different skeletal muscle chromatin states from the Roadmap Epigenomics Project,^27^ and (iii) in CCCTC‐binding factor (CTCF) and enhancer of zeste homologue 2 (EZH2) transcription factors binding sites in HSMMtube from the ENCODE project. CTCF is a multifunctional protein involved in gene regulation and chromatin organization,^28^ while EZH2 is the functional enzymatic component of the Polycomb repressive complex 2 (PRC2).^29^ A *P*‐value <0.005 was deemed significant.

We performed gene ontology (GO), KEGG, and Reactome enrichment on the age‐related DMRs using all tested CpGs as the background (i.e. the 649 250 CpGs included in the meta‐analysis), thanks to the goregion function from the *missMethyl* package.^30^ We used our own improved annotation of the epigenome and largely based on the comprehensive annotation of Zhou *et al*. of Illumina HumanMethylation arrays^31^ as well as the chromatin states in skeletal muscle from the Roadmap Epigenomics Project,^27^ and the latest GeneHancer information.^32^ The goregion function accounts for the biased distribution of CpGs in genes.^33^ All GO, KEGG, and Reactome terms with FDR < 0.005 were deemed significant.^34^, ^35^ To make sense of the many GO terms obtained as output, we used REVIGO^36^ that clusters GO terms according to semantic similarity.

### Integration of methylome, transcriptome, and proteome changes with age

Each gene with at least one DMR annotated to it was considered a differentially methylated gene (DMG). To gain insights into the functional consequences of DNA methylation changes with age in skeletal muscle, we compared DMGs with known differentially expressed genes at the transcriptomic^37^ and proteomic^38^ levels with advancing age. A transcriptomic meta‐analysis in skeletal muscle was recently published,^39^ but it focused on exercise‐induced changes instead of age‐related changes. Thus, we used the transcriptomic meta‐analysis of age by Su *et al*. that combined 2852 public gene expression arrays in skeletal muscle and identified 957 genes whose mRNA levels changed with age.^37^ Ubaida‐Mohien *et al*. performed a large‐scale proteomics analysis of human skeletal muscle and identified 1265 genes whose protein levels were altered with age.^38^ We used a *χ* ^2^ test to see whether a disproportionate number of DMGs were also differentially expressed at the mRNA or protein level, and a *P*‐value <0.005 was deemed significant.

### Update of the muscle epigenetic clock (MEAT 2.0)

Since the development of the original muscle clock that used 682 samples from 12 datasets to predict age from DNA methylation data,^9^ we gathered additional 371 samples from five datasets (+159 from Gene SMART, +65 from ABOS, +42 from LITER, +57 from GSE135063, and +48 from EPIK). We therefore updated the clock with these new samples, using the same algorithm and methodology.^9^ Briefly, we first preprocessed each dataset separately (i.e. probe/sample filtering, adjustment of type I and type II probes, and correction for batch effects); then, we reduced each dataset to all the CpGs that were common between them (18 747 CpGs). To obtain DNA methylation profiles that were comparable between datasets, we calibrated each dataset to GSE50498 using an adapted version of the BMIQ algorithm.^9^ We then used elastic net regression on a transformed version of age to create the new muscle clock (MEAT 2.0).^9^ Finally, given the limited number of datasets and the biased age distribution in each dataset, we estimated the accuracy of the new muscle clock in an unbiased manner using a leave‐one‐dataset‐out cross‐validation procedure, as described in our original paper.^9^

## Results

### Widespread age‐related DNA methylation changes at genes involved in skeletal muscle structure, development, and function

**Figure 1:**
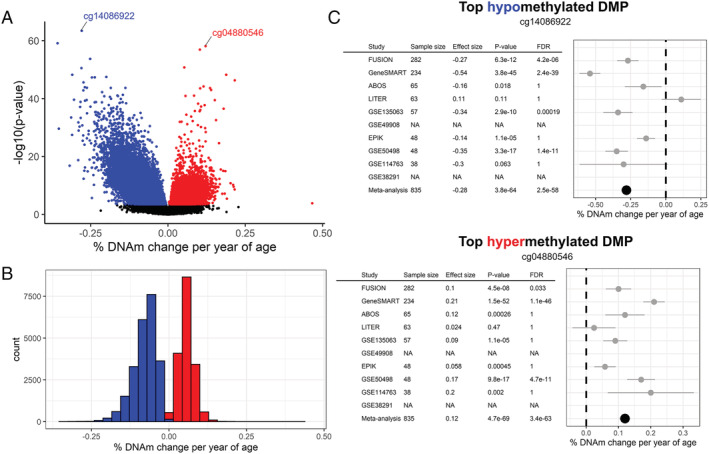
Age‐related DNA methylation loci in human skeletal muscle. *(A)* Meta‐analysis effect size (*x*‐axis) and meta‐analysis significance (*y*‐axis) for the 649 250 tested CpGs. Hypomethylated (blue) and hypermethylated (red) points represent differentially methylated position (DMPs) at a false discovery rate (FDR) <0.005. *(B)* Distribution of age‐related DNA methylation change at hypo‐DMPs and hyper‐DMPs. *(C)* Forest plots of the top hypomethylated and hypermethylated DMPs, showing sample size, effect size, *P*‐value, and FDR for each individual study as well as their meta‐analysis. Studies with missing information (‘NA’) mean that this CpG was not analysed in the dataset.

We first conducted an EWAS meta‐analysis of age in skeletal muscle using 10 datasets (total *n* = 908 samples from 601 individuals, *Table* 1) and uncovered a small, widespread effect of ageing on the skeletal muscle epigenome. Six per cent of all tested CpGs were associated with age in skeletal muscle (40 479 DMPs corresponding to 6710 DMRs, both at FDR < 0.005, *Figure* 1A and *Tables* S2 and S3). We found slightly more hypomethylated than hypermethylated DMPs (57% hypo‐DMPs and 43% hyper‐DMPs, *Table* S2). The magnitude of age‐related DNA methylation changes was small and similar for both hypo‐DMPs and hyper‐DMPs: hypo‐DMPs lost an average of ~0.8% in methylation per decade of life, and hyper‐DMPs gained an average of ~0.6% in methylation per decade of life (*Figure* 1B).

**Table 1:** Characteristics of the 10 cohorts included in the EWAS meta‐analysis of age

Dataset ID	Array	Number of unique individuals	Number of samples	Health status	Age (mean ± SD)	Age range (min–max)	% male	Ethnicity
FUSION	HMEPIC	282	282	Healthy/T2D	59.4 ± 7.9	20–77	54%	Caucasian
Gene SMART	HMEPIC	66	234	Healthy	32 ± 8.1	18–45	80%	Caucasian + one mixed Aboriginal/Caucasian
ABOS	HM450	65	65	Lean/obese/obese with T2D	44 ± 8.2	23–61	0%	Caucasian
LITER	HMEPIC	21	63	Healthy	26.0 ± 5.9	20–39	100%	75% Caucasian, 16% Asian, 8% mixed
GSE135063	HMEPIC	24	57	Healthy/obese	38.9 ± 10	23–58	100%	Caucasian
GSE49908	HM27	51	51	Healthy	50 ± 17	21–77	100%	Caucasian
GSE50498	HM450	48	48	Healthy	47 ± 26	18–89	100%	Caucasian
EPIK	HMEPIC	14	48	Healthy	45.4 ± 22.3	20–71	100%	Caucasian
GSE114763	HMEPIC	8	38	Healthy	29 ± 6	19–39	100%	Caucasian
GSE38291	HM27	22	22	Healthy/T2D (twins)	68 ± 8	53–80	45%	Caucasian

EWAS, epigenome‐wide association study; SD, standard deviation; T2D, type 2 diabetes.

The number of samples can differ from the number of unique individuals if the same individuals have been profiled for DNA methylation patterns multiple times, such as before and after exercise training.

Each dataset had a unique study design that required adjustment for factors that are known to affect DNA methylation levels, such as sex,^40^ BMI,^41^ and T2D.^42^ We adjusted each dataset for these factors, but we noted that age was associated with BMI or T2D in some datasets (*Table* S1). For example, older individuals from the GSE50498 dataset had a higher BMI than younger individuals (4.1 kg/m^2^ heavier, *P* = 0.0011), so it is possible that the age‐related signal captured in this dataset was partially confounded by BMI. We repeated the meta‐analysis without GSE50498, but results were largely unchanged (*Figure* S3a). We also repeated the meta‐analysis excluding T2D patients from the FUSION, ABOS, and GSE38291 datasets, but results remained unchanged (*Figure* S3b). We also repeated the meta‐analysis without the ABOS dataset whose muscle of origin differed from that of the other datasets (rectus abdominis vs. vastus lateralis muscle). However, results remained unchanged (*Figure* S3c). Finally, we repeated the meta‐analysis omitting eight non‐Caucasian individuals from the Gene SMART and LITER cohorts. However, results remained unchanged (*Figure* S3d). This confirms that our results are not confounded by BMI, T2D, the type of skeletal muscle, or the presence of a few ethnically diverse individuals.

**Figure 2:**
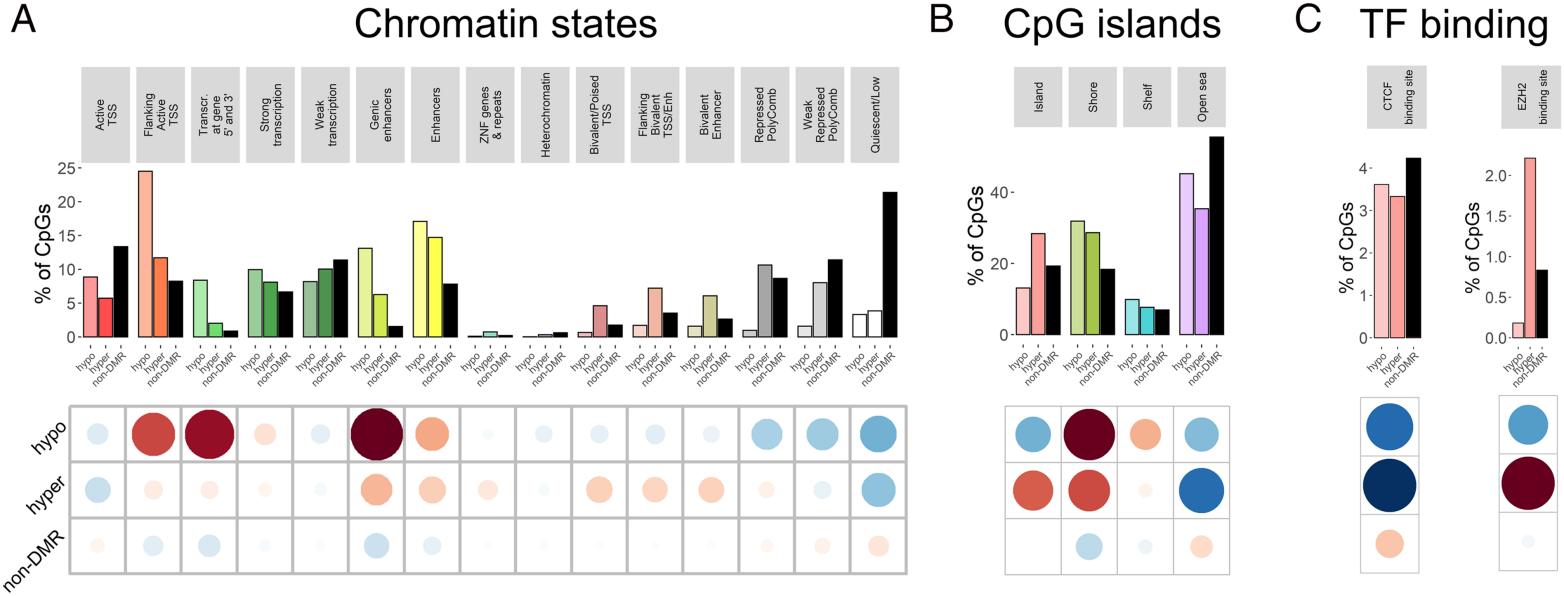
Distribution of hypomethylated and hypermethylated differentially methylated regions (DMRs) and non‐DMRs in functional regions of the genome. *(A)* Distribution in chromatin states from male skeletal muscle from the Roadmap Epigenomics Project^27^; *(B)* distribution with respect to CpG islands, shore = ±2 kb from the CpG island, shelf = ±2–4 kb from the CpG island, and open sea ≥4 kb from a CpG island; and *(C)* distribution in CCCTC‐binding factor (CTCF) and enhancer of zeste homologue 2 (EZH2) binding sites in skeletal muscle myotubes differentiated from the HSMM cell line (HSMMtube) from the ENCODE project. The grids under the figures represent the residuals from the *χ* ^2^ test, with the size of the circles being proportional to the cell's contribution; red indicates an enrichment of the DMR category in the functional region, while blue indicates a depletion of the DMR category in the functional region.

We then focused on the DMRs for all downstream analyses, as DMRs remove spatial redundancy (CpG sites within ~500 bp are typically highly correlated^24^), and they may provide more robust and functionally important information than DMPs.^25^, ^26^ As with DMPs, we found slightly more hypomethylated than hypermethylated DMRs (61% hypo‐DMRs and 39% hyper‐DMRs, *Table* S3). DMRs' distribution in chromatin states was different from that of all tested CpGs (*χ* ^2^ test *P*‐value <2.2 × 10^−16^, *Figure* 2). DMRs were strongly under‐represented in quiescent regions, while over‐represented at enhancers and around active transcription start sites (TSSs). However, hypo‐DMRs were more strongly over‐represented in genic enhancers and around active TSSs; conversely, only hyper‐DMRs showed over‐representation in and around bivalent enhancers and promoters, and in regions actively repressed by PolyComb proteins. The distribution of hyper‐DMRs and hypo‐DMRs also varied with respect to CpG islands: both were under‐represented in open seas and over‐represented in CpGs island shores, but only hyper‐DMRs were over‐represented in CpG islands (*χ* ^2^ test *P*‐value <2.2 × 10^−16^, *Figure* 2). Finally, both hypo‐DMRs and hyper‐DMRs were under‐represented in CTCF binding sites in differentiated skeletal muscle myotubes, but only hyper‐DMRs were strongly over‐represented in EZH2 binding sites (*Figure* 2).

**Figure 3:**
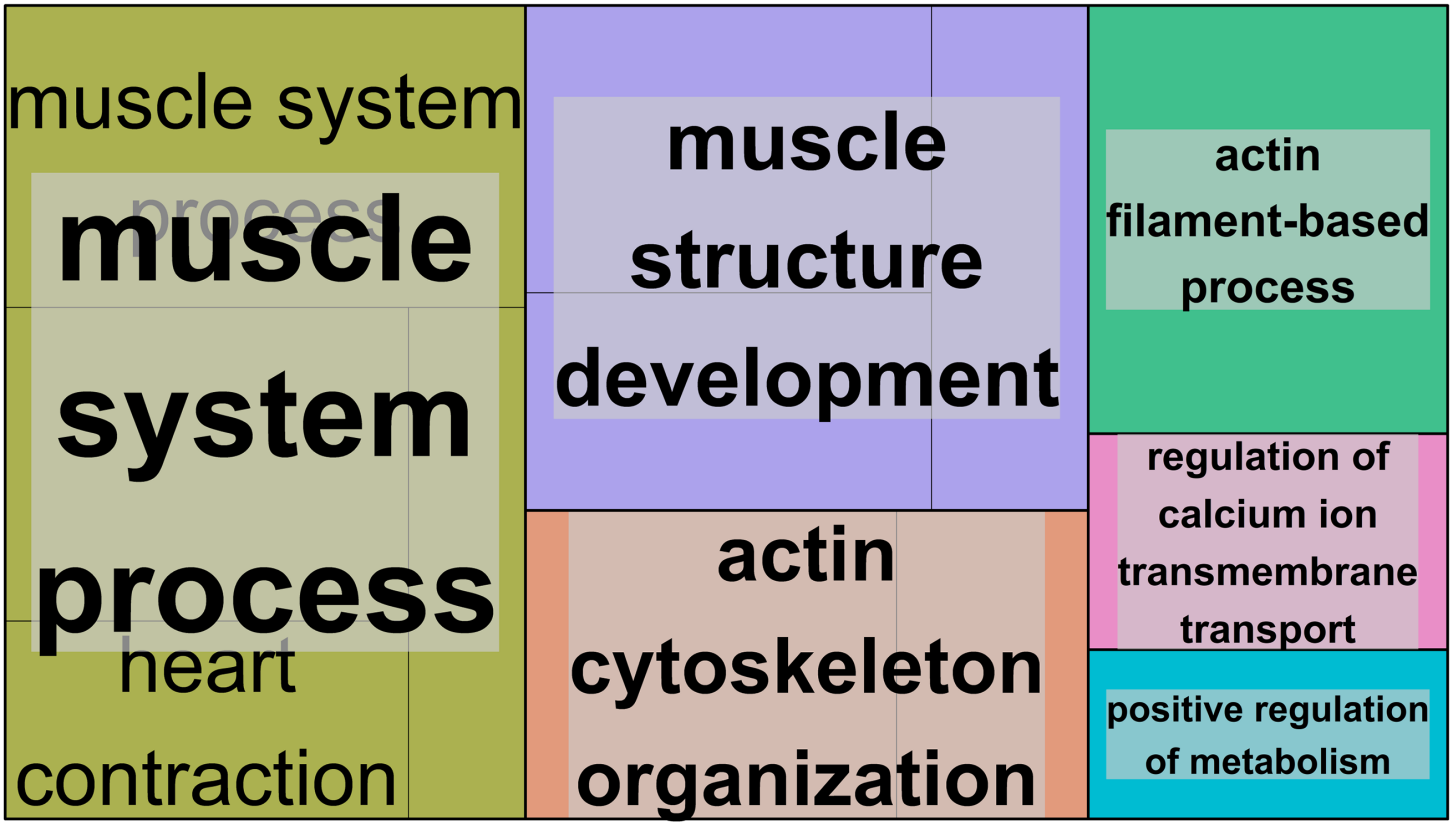
Gene set enrichment analysis of the differentially methylated genes. This treemap shows the clustering of the 48 significant gene ontology (GO) terms belonging to the ‘biological processes’ category. The 48 GO terms were clustered based on semantic similarity measures using REVIGO,^36^ with each rectangle corresponding to a single cluster representative. The representatives are joined into ‘superclusters’ of loosely related terms, visualized with different colours. The size of the rectangles is proportional to the –log_10_(*P*‐value) of the GO term.

Next, we integrated a comprehensive annotation of Illumina HumanMethylation arrays^31^ with chromatin states from the Roadmap Epigenomics Project^27^ and the latest GeneHancer information^32^ to map the DMRs to genes (*Table* S3). Including non‐coding genes, there were 6367 genes that harboured at least one DMR, hereinafter referred to as DMGs. A pathway enrichment on the DMRs revealed that DMGs were enriched for 48 GO terms (*Table* S4), all of which related to skeletal muscle structure development, muscle contraction, and calcium transporter regulation (*Figure* 3). In agreement with this, we also found enrichment for the KEGG term ‘cardiac muscle contraction’ (FDR = 0.0038) and for the Reactome term ‘muscle contraction’ (FDR = 0.00020). Of note, a GSEA enrichment restricted to the hypomethylated DMGs yielded very similar results (*Table* S5), but no significant enrichment was found for hypermethylated DMGs.

### Differentially methylated genes are enriched for genes showing age‐related changes at the mRNA and protein levels

**Figure 4:**
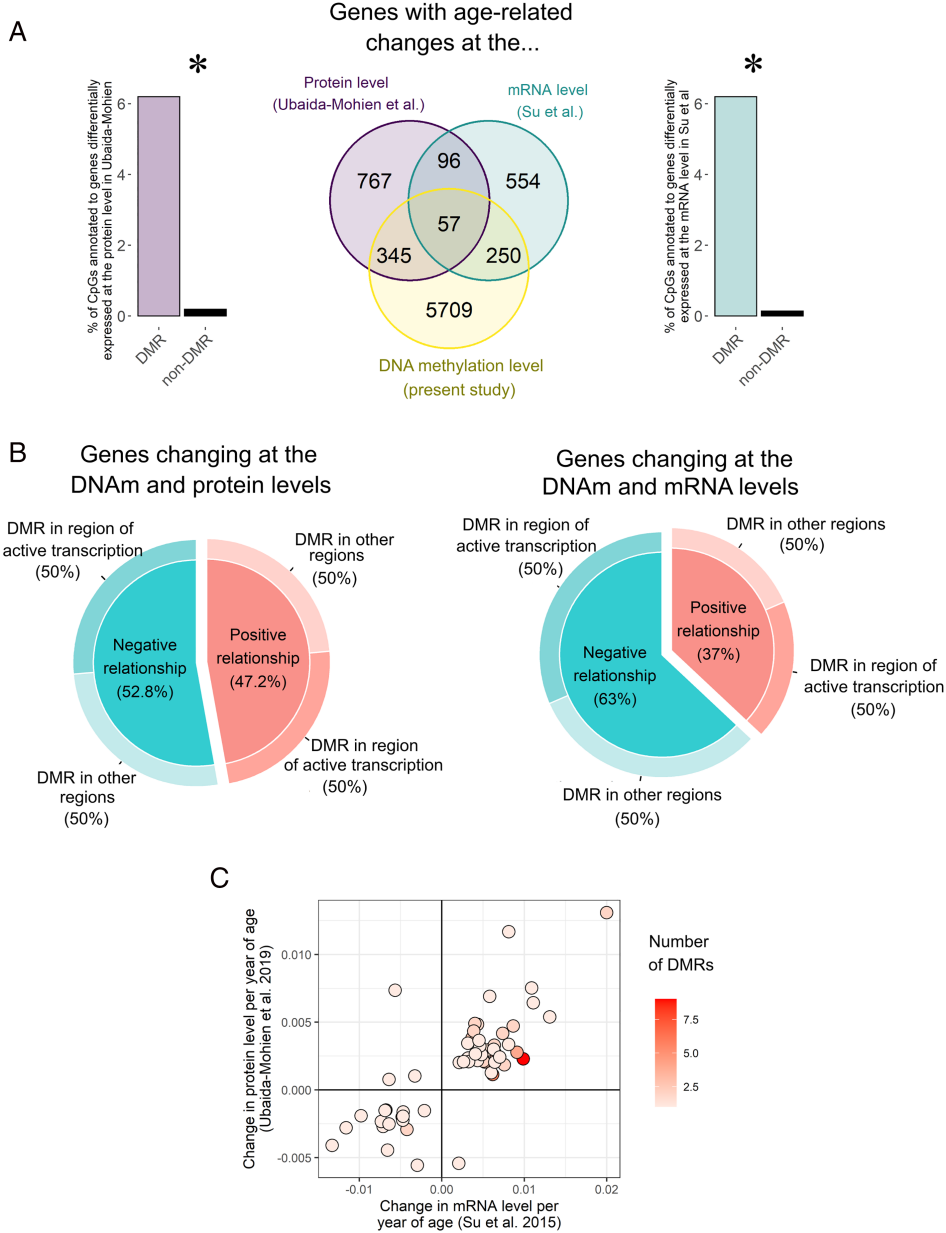
Integration of DNA methylation, and mRNA and protein changes with age in human skeletal muscle. *(A)* Overlap between genes that change with age at the DNA methylation level (yellow, present study), mRNA level (green, Su *et al*.^37^), and protein level (purple, Ubaida‐Mohien *et al*.^38^). On each side of the Venn diagram, we showed the distribution of differentially expressed genes among the differentially methylated genes (DMGs) and the non‐differentially methylated genes (non‐DMGs). *χ ^2^ test *P*‐value <0.005. *(B)* Relationship between age‐related DNA methylation changes and mRNA changes (right) or protein changes (left): ‘negative relationship’ means that a gene that was up‐regulated with age at the gene expression level showed lower DNA methylation with age in the present study, and a gene that was down‐regulated with age at the gene expression level showed higher DNA methylation with age in the present study. As the relationship between DNA methylation and gene expression differs depending on the genomic context, we further split the age‐related DNA methylation changes between those located in regions of active transcription and those located in other regions. *(C)* Scatter plot showing the change in mRNA (*x*‐axis) and protein (*y*‐axis) per year of age for the 57 genes altered at all three omics levels. Each gene was coloured according to the number of DMRs annotated to it, from 1–3 DMRs for most genes all the way up to 9 DMRs. Naturally, longer genes (e.g. NXN and ABLIM2) have a greater propensity to have more DMRs given their high numbers of CpGs.

We investigated the potential downstream effects of these age‐related DNA methylation changes on mRNA and protein expression in muscle. We utilized two external published studies: a transcriptomic meta‐analysis of age that combined 2852 public gene expression arrays in skeletal muscle^37^ and a large‐scale proteomic analysis of age in skeletal muscle from 58 healthy individuals aged 20–87 years.^38^ Su *et al*.^37^ identified 957 genes whose mRNA levels change with age, and Ubaida‐Mohien *et al*.^38^ identified 1265 genes whose protein levels change with age. Forty‐one per cent of the genes whose mRNA levels change with age were also altered at the DNA methylation level, and 42% of the genes whose protein levels change with age were also altered at the DNA methylation level (*Figure* 4A). Furthermore, the DMGs included proportionally many more differentially expressed genes than the non‐DMGs (*χ* ^2^ test *P*‐value <2.2 × 10^−16^, *Figure* 4A), indicating that such a large overlap between differential DNA methylation and differential gene expression with age cannot be attributed to chance alone.

Next, we investigated in more details the relationship between DNA methylation and mRNA or protein expression. This relationship is complex and depends on the genomic context, particularly the underlying chromatin state^43^; an increase in DNA methylation is usually associated with a down‐regulation of gene expression, but the opposite pattern is observed in gene bodies of actively transcribed genes. We found that the relationship between DNA methylation and mRNA expression was negative in only 63% of cases, regardless of whether the DMR was in a gene body or not, and the relationship between DNA methylation and protein expression did not show any predominant pattern (*Figure* 4B). Fifty‐seven genes were altered at all three omic levels (*Table* S6, *Figure* 4C). There was a high concordance between the transcriptomic and proteomic studies: an age‐related increase in mRNA level was most often mirrored by an age‐related increase in protein level and vice versa (*Figure* 4C).

We also looked at age‐related DNA methylation changes in light of age‐related physiological changes in muscle, namely, muscle atrophy,^44^ alterations in lipid metabolism,^45^ and increase in the proportion of hybrid muscle fibres (type IIx).^46^ We focused on DNA methylation, mRNA expression, and protein expression changes at genes known to promote muscle atrophy (*FBXO32*, *TRIM63*, *MYOG*, *HDAC4*, and *HDAC5*),^47^ involved in fatty acid metabolism in muscle (*CD36*, *GOT2*, *CPT1A*, *HADH*, *LPL*, *SLC27A1*, *SLC27A4*, and *UCP3*),^48^, ^49^, ^50^, ^51^ and encoding myosin light and heavy chains that discriminate type I, type IIa, and type IIx fibres (*MYH6*, *MYH7*, *MYH1*, *MYL3*, and *MYH2*).^52^ While only three lipid metabolism genes were DMGs, with no corresponding changes in gene expression, all genes promoting muscle atrophy were hypomethylated with increased age. In particular, *HDAC4* was mostly hypomethylated, and there was a corresponding increase in mRNA levels (*Table* 2), and atrogin‐1 was also hypomethylated, with a corresponding increase in protein levels (*Table* 2). Nearly all genes encoding the myosin chains were hypomethylated, but no gene expression changes were detected (*Table* 2).

**Table 2:** Age‐related epigenetic, transcriptomic, and proteomic changes at candidate genes involved in skeletal muscle atrophy, lipid metabolism, and fibre‐type specification

	Gene name	Gene symbol	Number of DMRs	DNA methylation change with age	Gene expression change with age
Muscle atrophy	Atrogin‐1	*FBXO32*	2	Hypomethylation	Increased protein expression
	MuRF1	*TRIM63*	3	Hypomethylation	
	Myogenin	*MYOG*	1	Hypomethylation	
	Histone deacetylase 4	*HDAC4*	19	Hypo and hypermethylation	Increased mRNA expression
	Histone deacetylase 5	*HDAC5*	2	Hypomethylation	
Fatty acid metabolism	Fatty acid translocase	*CD36*	0		
	Plasma membrane fatty acid binding protein	*GOT2*	0		Decreased mRNA and protein expression
	Carnitine palmitoyltransferase I	*CPT1A*	0		
	β‐Hydroxyacyl‐CoA dehydrogenase	*HADH*	2	Hypomethylation	
	Lipoprotein lipase	*LPL*	1	Hypomethylation	
	Long‐chain fatty acid transport protein 1	*SLC27A1*	0		
	Long‐chain fatty acid transport protein 4	*SLC27A4*	0		Decreased protein expression
	Uncoupling protein 3	*UCP3*	1	Hypomethylation	
Fibre type‐specific genes	Myosin heavy chain 2	*MYH2*	1	Hypomethylation	
	Myosin heavy chain 1	*MYH1*	0		
	Myosin light chain 3	*MYL3*	2	Hypomethylation	
	Myosin heavy chain 6	*MYH6*	1	Hypomethylation	
	Myosin heavy chain 7	*MYH7*	5	Hypomethylation	

DMR, differentially methylated region.

DNA methylation changes are from the present EWAS meta‐analysis, mRNA changes are from Su *et al*.,^37^ and protein changes are from Ubaida‐Mohien *et al*.^38^

**Figure 5:**
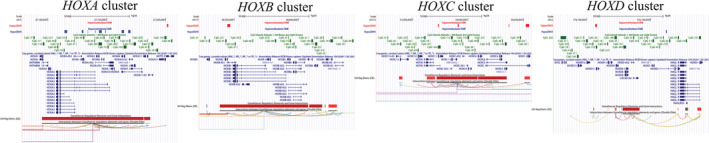
Genome browser view (hg38) of differential DNA methylation at the four HOX gene clusters. Tracks, from top to bottom, correspond to hypermethylated and hypomethylated DMRs in the present meta‐analysis, CpG islands, genes from RefSeq, and GeneHancer regulatory elements and interactions.

We also compared our DMPs with CpGs associated with age in two of the individual studies used in our meta‐analysis^7^, ^8^ to confirm and validate genes and regions. We found that half of the DMPs discovered by Zykovich *et al*.^7^ and 60% of the DMPs discovered by Day *et al*.^8^ were validated by our meta‐analysis. For instance, we confirmed the widespread intragenic hypermethylation of *TBCD* ^7^ and *NFATC1*.^7^ Such a large overlap is not surprising given that both studies were included in the meta‐analysis. For unbiased replication, we compared our DMPs with CpGs associated with age in a recent, independent study.^11^ Only 7% of the DMPs identified by Turner *et al*.^11^ were replicated in our meta‐analysis, but 99% of them were consistently hypomethylated or hypermethylated with age. As reported by Turner *et al*.,^11^ we also found a systematic alteration of all *HOX* gene clusters (*HOXA*, *HOXB*, *HOXC*, and *HOXD*), but not necessarily the same *HOX* genes or in the same direction.^11^ We detected nine DMRs in the *HOXA* cluster that were nearly all hypomethylated, one hypermethylated DMR at *HOXB2*/*HOXB‐AS1*, four hypermethylated DMRs in the *HOXC* cluster, and two hypermethylated DMRs in the *HOXD* cluster (*Figure* 5).

### *MetaMeth*: an online tool to visualize the ageing profile of human skeletal muscle

We have made the results of the EWAS meta‐analysis of age in skeletal muscle available as an online webtool called *MetaMeth* (https://sarah‐voisin.shinyapps.io/MetaMeth/). The home page of the website provides a detailed list of instructions on how to visualize results and focus on specific CpGs, genes, or genomic regions of interest in a user‐friendly, interactive manner. To obtain forest plots for individual CpGs, users can enter the name of their CpG of interest (e.g. ‘cg11109027’) in the ‘Forest Plot’ tab, and the corresponding graph will appear, with the possibility to download the plot in jpg, png, or tif formats and at any resolution. To help with choosing CpGs to display, users can filter the list of CpGs based on their genomic location (e.g. genomic region, annotated gene, position with respect to CpG islands, chromatin states in male and female skeletal muscle, and TF binding). To download summary statistics for DMPs or DMRs in a table format, users can go to the ‘Summary Tables’ tab and download the data as an excel or csv file, after optionally filtering data based on genomic location and statistics. Finally, we have also displayed the scatter plot of genes showing methylation, and mRNA and protein changes with age as an interactive graph: users simply need to hover their mouse on one point of the graph to be shown the name of the gene and the number of DMRs annotated to it. The code used to produce the website is available in open access on Sarah Voisin's GitHub account (https://github.com/sarah‐voisin/MetaMeth).

### More samples in the muscle epigenetic clock do not change age prediction accuracy

**Figure 6:**
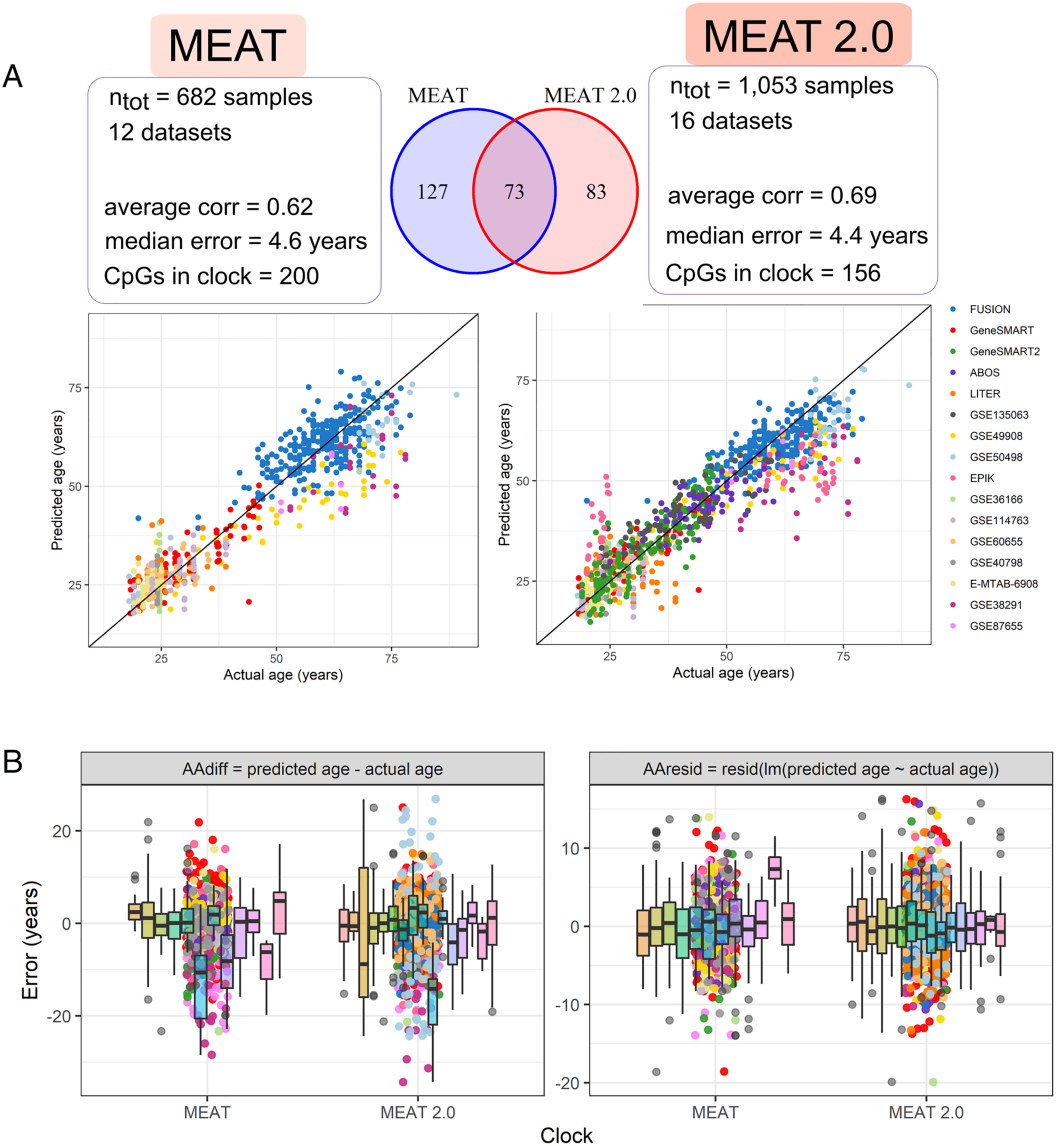
Original and new version of the muscle clock (MEAT). *(A)* Original (left, MEAT) and new version (right, MEAT 2.0) of the muscle clock.^9^ The Venn diagram represents the number of CpGs included in each clock and the number of CpGs in common between the two clocks. The graphs show predicted (*y*‐axis) against actual (*x*‐axis) age for each sample in the 16 datasets used to build the clocks. A leave‐one‐dataset‐out cross‐validation (LOOCV) procedure was used to obtain predicted age for a given dataset in an unbiased manner (16 LOOCV were performed, one per dataset). The summary statistics reported on the left‐hand side are the average correlation between actual and predicted age across datasets, the median absolute error in age prediction across datasets, and the number of CpGs automatically selected by the algorithm to build the clock. *(B)* Error in age prediction either as the difference between predicted and actual age (left panel) or as the residuals from a linear model of predicted against actual age (right panel). Note that both panels are on different scales.

The present EWAS meta‐analysis of age utilized all of the datasets included in the original muscle epigenetic clock (MEAT) that we recently published, with the exception of datasets that were invariant in age and the datasets that were too small (*n* < 20) (see Methods).^9^ The present study included an additional 371 samples from five datasets. Using the same algorithm and methodology, we updated the muscle clock with these new samples, reaching a total of *n* = 1053 human skeletal muscle samples from 16 datasets. The updated version of the clock (MEAT 2.0) uses DNA methylation at 156 CpGs to predict age, 73 of which were in common with MEAT (*Figure* 6A). We found that MEAT 2.0 only slightly outperforms MEAT, with an average Pearson correlation coefficient of 0.69 across datasets (vs. 0.62 for MEAT^9^) and a median error of only 4.4 years across datasets (vs. 4.6 years for MEAT^9^) (*Figure* 6B).

## Discussion

To paint a comprehensive picture of age‐related DNA methylation changes in human skeletal muscle, we conducted an EWAS meta‐analysis of age in human muscle across the lifespan, combining 908 samples from 10 independent datasets. In this study, we were able to demonstrate a profound effect of age on the muscle methylome. Additionally, we have provided a detailed account of the genomic context of age‐affected regions, reported putatively affected pathways, and integrated methylome changes with known transcriptome and proteome changes in muscle. To maximize the usefulness of this large‐scale EWAS meta‐analysis to the scientific community, we created a website named MetaMeth (https://sarah‐voisin.shinyapps.io/MetaMeth/), which allows researchers to visualize results in an interactive and user‐friendly manner. Finally, we updated our muscle clock^9^ with 371 newly acquired DNA methylation samples and found that the original version of the clock was already at optimal prediction accuracy.

Previous studies on the overall pattern of age‐related DNA methylation changes in muscle showed mixed results, three reporting more hypermethylation with age^7^, ^8^, ^11^ and one finding slightly more hypomethylation with age.^10^ We included three of these studies (GSE49908, GSE50498, and EPIK) in our meta‐analysis and found balanced amounts of hypomethylation and hypermethylation. Differences in coverage between studies are unlikely to explain the discrepancy in results, because the three HumanMethylation arrays were represented in these studies (27k, 450k, and 850k). It is more likely that the overall direction of age‐related DNA methylation change became more nuanced once these small‐scale studies were combined with the other nine datasets. This highlights the advantage of the meta‐analysis approach we utilized in identifying robust ageing‐related CpG sites across multiple, potentially conflicting studies. We detected thousands of age‐related DMRs, likely thanks to the unprecedented power achieved with 908 human muscle samples. We found limited but highly consistent overlap between age‐related changes identified in our meta‐analysis and those recently identified in a small, independent study.^11^ In particular, we validated age‐related changes in all *HOX* gene clusters. This is intriguing as epigenetic and transcriptomic alterations of all *HOX* clusters were recently reported in a mouse model of accelerated ageing,^53^ suggesting that a dysregulation of developmental genes controlling cell identity underpins mammalian ageing. Additionally, we found hypomethylation at genes promoting muscle atrophy, mirrored by an increase in gene expression for *HDAC4* and an increase in protein expression for *atrogin‐1*.

Age‐affected regions were not randomly spread across the genome and were particularly abundant around active TSS regions and in enhancers. Furthermore, hypomethylated and hypermethylated regions showed a distinct distribution largely consistent with previous reports on ageing; during ageing, DNA methylation tends to increase at Polycomb target genes^54^, ^55^ and bivalent chromatin domains,^55^, ^56^ while decreasing at enhancers in both mice and humans.^54^, ^55^ To explain the age‐related hypermethylation of Polycomb target genes, Jung and Pfeifer proposed a mechanism involving competition between Polycomb complexes and DNA methyltransferase 3 (DNMT3)^57^: the ability of the Polycomb machinery to target unmethylated CpG‐rich target sequences erodes with age, leaving room for DNMT3 to bind and slowly methylate Polycomb target genes over time, potentially leading to reduced plasticity of the hypermethylated genes. This was entirely consistent with our findings: hypermethylated DMRs were strongly enriched in CpG islands and EZH2 binding sites (EZH2 is the enzymatic subunit of the Polycomb complex). Polycomb target genes and bivalent chromatin domains are linked to developmental and differentiation processes,^54^ which corroborated the pathway enrichment showing numerous GO terms related to muscle cell differentiation and skeletal muscle development. Neither the root nor the functional consequences of enhancer hypomethylation are known, but it may stem from altered DNMT and TET enzymes activity and might lead to activation of cryptic transcripts or disrupt enhancer–gene interactions.^54^ Taken together, our findings indicate a widespread effect of age on DNA methylation levels in skeletal muscle at genes fundamental for skeletal muscle development, structure, and differentiation.

It is challenging to speculate regarding the consequences of DNA methylation changes on gene expression, as both hypomethylation and hypermethylation have been associated with increased gene expression,^58^, ^59^, ^60^ likely depending on the genomic context (i.e. CpG density, location with respect to promoter/first exon/gene body/enhancer). In addition, ~8% of DMGs harboured both hypermethylated and hypomethylated DMRs, further complicating the interpretation of DNA methylation changes. We suggest that DNA methylation changes likely reflect changes in gene activity, but the directionality is unclear. This is consistent with our integration of the present EWAS meta‐analysis of age with two large, published transcriptomic and proteomic studies of age in human skeletal muscle.^37^, ^38^ Genes altered at the DNA methylation level were much more likely to be altered at the transcriptomic and proteomic levels. However, the relationship between DNA methylation and gene expression was negative only ~50–60% of the time. We could not assess whether age‐related DNA methylation changes are a cause or a consequence of age‐related gene expression changes, but the two scenarios are not mutually exclusive. We also noted that age‐related mRNA and protein changes in skeletal muscle were highly consistent, as there was a strong positive correlation between mRNA and protein changes with age in human skeletal muscle. This reinforces the utility of large‐scale studies, including meta‐analyses, to produce robust, replicable results identifying DNA methylation targets. Future studies should explore the origin and functional consequences of these age‐related omic changes in human skeletal muscle and investigate whether the cause of the ageing processes is similar across tissues. As changes in the epigenetic landscape are one of the primary hallmarks of ageing, understanding its origin would narrow down our focus on putative genetic or/and epigenetic regions, with the ultimate goal of targeting them with lifestyle or pharmacological interventions to slow down the ageing process at the molecular level. Future studies should aim to find interventions easily accessible to a wide range of people, such as exercise training or dietary interventions, to slow down, or perhaps even reverse, age‐related epigenetic changes in skeletal muscle.

Recently, we established an epigenetic clock for human skeletal muscle, using 682 samples from 12 datasets.^9^ Here we updated this clock (MEAT 2.0) by using 1053 samples from 16 datasets, particularly adding more female and middle‐aged individuals that were under‐represented in MEAT. MEAT 2.0 automatically selected 205 CpGs for age prediction, only 98 of which were in common with the CpGs selected by MEAT. While such a small overlap may seem surprising, it likely stems from the machine learning algorithm underlying the clocks: tens of thousands of CpGs change with age, but only a handful of CpGs are selected by the elastic net model, so this group of CpGs is only one of the many possible combinations of CpGs that can predict age with high accuracy.^6^ We tested whether the accuracy of the muscle clock is improved by feeding more samples to the machine learning algorithm. Surprisingly, the accuracy of the new version of the clock barely improved, from 0.62 to 0.66 in average correlation between predicted and actual age and from 4.6 to 4.5 years in median error in age prediction. This suggests that the original muscle clock was already sufficiently accurate for age prediction in human skeletal muscle using the Illumina HumanMethylation array technology. We have however updated the R package *MEAT* on Bioconductor with this new clock, providing users the possibility to choose between the original version (MEAT) and updated version (MEAT 2.0) of the clock for their analyses.

The age‐related changes in the muscle methylome uncovered herein and the epigenetic age calculated from the MEAT clock reflect both intracellular changes in methylation levels and age‐related changes in muscle cell‐type composition. Older muscle tends to have a greater proportion of type IIx (hybrid) muscle fibres,^46^ shows fat^61^ and macrophage^62^ infiltration, and displays lower numbers of satellite cells,^63^ which can alter the methylome of bulk muscle tissue. However, we adjusted the analyses for bias and inflation^21^ to account for unmeasured factors such as population substructure, batch effects, and cellular heterogeneity.^22^ Uncovering the intracellular changes of different muscle cell types with age was beyond the scope of this study, and we did not have information on individual cellular profiles to answer this question. Nevertheless, the results shown here, along with the epigenetic clock and open‐access search engine we developed, may still be highly valuable to ageing researchers whose focus is unrelated to cell type‐specific ageing. It should also be noted that the conclusions of this study may not apply to the human population as a whole, as 98% of the samples were of Caucasian origin and 71% were from male subjects. Future studies should make efforts to profile the methylomes of under‐represented groups to provide a picture of ageing that reflects the world population.

To provide the scientific community with a tool to assess DNA methylation changes with age in skeletal muscle, we have created a user‐friendly, interactive, and transparent way to explore our results. We built a web‐based tool called *MetaMeth* (https://sarah‐voisin.shinyapps.io/MetaMeth/), largely inspired by the *MetaMex* tool developed by Pillon *et al*. for transcriptomic meta‐analysis of exercise training and inactivity in human skeletal muscle.^39^ Users are able to explore DMPs, DMRs, forest plots, and omics integration and to filter and download the results. This freely available website is likely to advance the field of ageing science as a whole.

## Author contributions

S.V. and N.E. contributed in the conceptualization of the study; S.V. and S.H. in the methodology; S.V. in the investigation; S.V. in the formal analysis; M.J., S.L., N.R.H., L.M.H., L.R.G., S.G., M.O., M.J., K.J.A., J.‐L.M.T., A.G., C.J., R.C., H.V., V.R., F.P., P.F., S.B., M.T., A.P.S., A.S., M.R., S.H., and N.E. in the resources; S.V. in the software; S.V. and N.E. in writing of the original draft; S.V., M.J., S.L., S.G., M.O., M.J., K.J.A., A.G., C.J., F.P., J.M.C., S.B., M.T., A.P.S., A.S., M.R., S.H., and N.E. in writing of the review and editing; and S.V., L.R.G., and N.E. in funding acquisition.

## Conflict of interest

None declared.

## Funding

We are grateful for the support of the Australian National Health and Medical Research Council (NHMRC) via S.V.'s Early Career Research Fellowship (APP11577321) and N.E.'s Career Development Fellowship (APP1140644). We are also grateful for the support of the Jack Brockoff Foundation via S.V.'s medical grant. We also thank the Australian Research Council (ARC) for supporting this study (DP190103081 and DP200101830). The Gene SMART and LITER studies were both supported by the Collaborative Research Network for Advancing Exercise and Sports Science (201202) from the Department of Education and Training, Australia. N.R.H. and Ms J.‐L.M.T. were supported by a PhD stipend also provided by Bond University CRN‐AESS. This research was also supported by infrastructure purchased with Australian Government EIF Super Science Funds as part of the Therapeutic Innovation Australia—Queensland Node project (L.R.G.). A.P.S. was supported by GlaxoSmithKline, North Staffordshire Medical Institute, the Society fort Endocrinology, the Medical Research Council (MRC) and Engineering and Physical Sciences Research Council (EPSRC), UK Doctoral Training Centre, and the Norwegian School of Sport Sciences (Norges Idrettshøgskole). The work was also supported by the German Federal Ministry of Education and Research [Bundesministerium für Bildung und Forschung (BMBF): DZD Grant 82DZD00302] and the Brandenburg State (Germany). The EPIK study was supported by the Foundation Scientific Research—Flanders (FWO Grant F.0898.15).

## Ethics approval and consent to participate

All studies used in this article^7^, ^8^, ^9^, ^10^, ^13^, ^14^, ^15^, ^16^, ^17^, ^18^ received approval of their respective ethics committees and complied with the Declaration of Helsinki.

## Supporting information


**Figure S1.** Age distribution in each of the 10 datasets included in the EWAS metahyphen;analysis, and database of origin. dbGAP = database of Genotypes and Phenotypes; GEO = Gene Expression Omnibus.

**Figure S2.** Quantile‐quantile plot of −log10 transformed P‐values for each of the 10 datasets included in the EWAS meta‐analysis. Right panel using uncorrected P‐values and left panel using bacon bias‐ and inflation‐corrected P‐values.

**Figure S3.** Comparison of results from the full meta‐analysis and from a meta‐analysis excluding GSE50498 (a), type 2 diabetes (T2D) patients (b), the ABOS cohort (c), or non‐Caucasian individuals (d). Each point is one of the 40,479 differentially methylated positions (DMPs) discovered in the full meta‐analysis. To compare results from the full and partial meta‐analyses, we plotted the effect size in the full meta‐analysis (x‐axis), against the effect size in the partial meta‐analysis (y‐axis). To show whether DMPs remained significant in the partial meta‐analysis, we coloured points according to the false discovery rate (FDR) in the partial meta‐analysis.

**Table S1.** Supporting information

## Data Availability

The GSE151407, GSE49908, GSE50498, GSE114763, GSE38291, and GSE135063 are available in the Gene Expression Omnibus repository (https://www.ncbi.nlm.nih.gov/geo). The FUSION study is available on the dbGaP repository (https://www.ncbi.nlm.nih.gov/gap/). The ABOS and EPIK studies datasets are available from the corresponding author on reasonable request. The online webtool (*MetaMeth*) generated during the current study is available at https://sarah‐voisin.shinyapps.io/MetaMeth/?_ga=2.176854501.1513337947.1598765391‐1534254090.1593032027. The source code for *MetaMeth* is available at https://github.com/sarah‐voisin/MetaMeth.
