# Supplementary figures and images for: Meta‐analysis of genome‐wide DNA methylation and integrative omics of age in human skeletal muscle
Source: J Cachexia Sarcopenia Muscle. 2021 Jun 30;12(4):1064–78. doi: 10.1002/jcsm.12741 (PMC8350206; doi:10.1002/jcsm.12741)

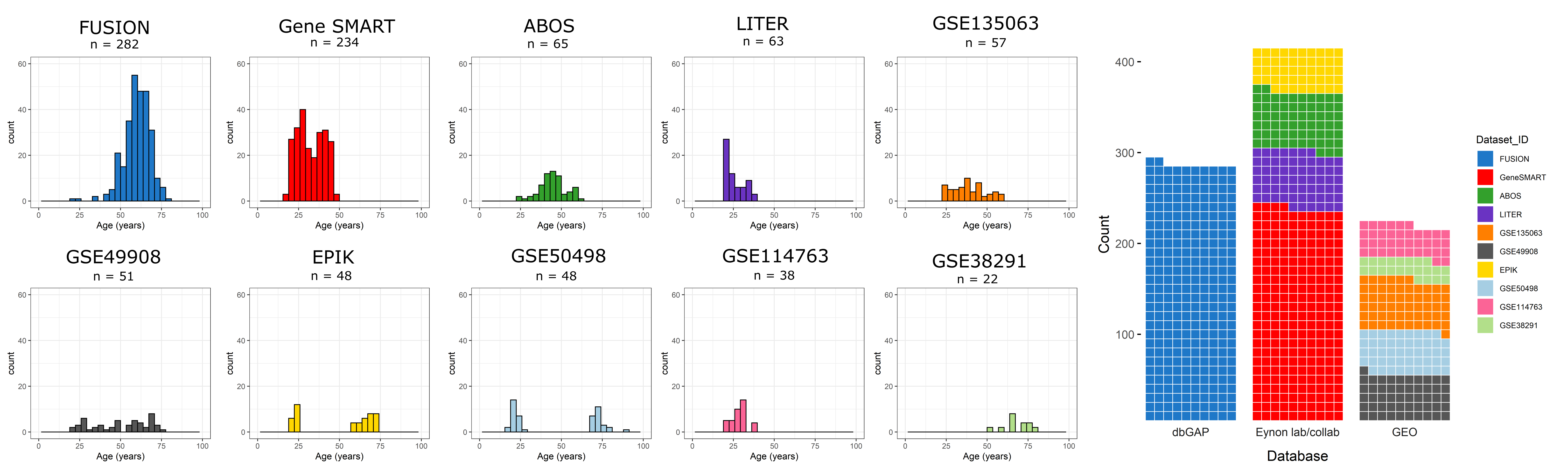

Supplement: Supplementary file 1 — Figure S1. Age distribution in each of the 10 datasets included in the EWAS metahyphen;analysis, and database of origin. dbGAP = database of Genotypes and Phenotypes; GEO = Gene Expression Omnibus. [file JCSM-12-1064-s003.png]

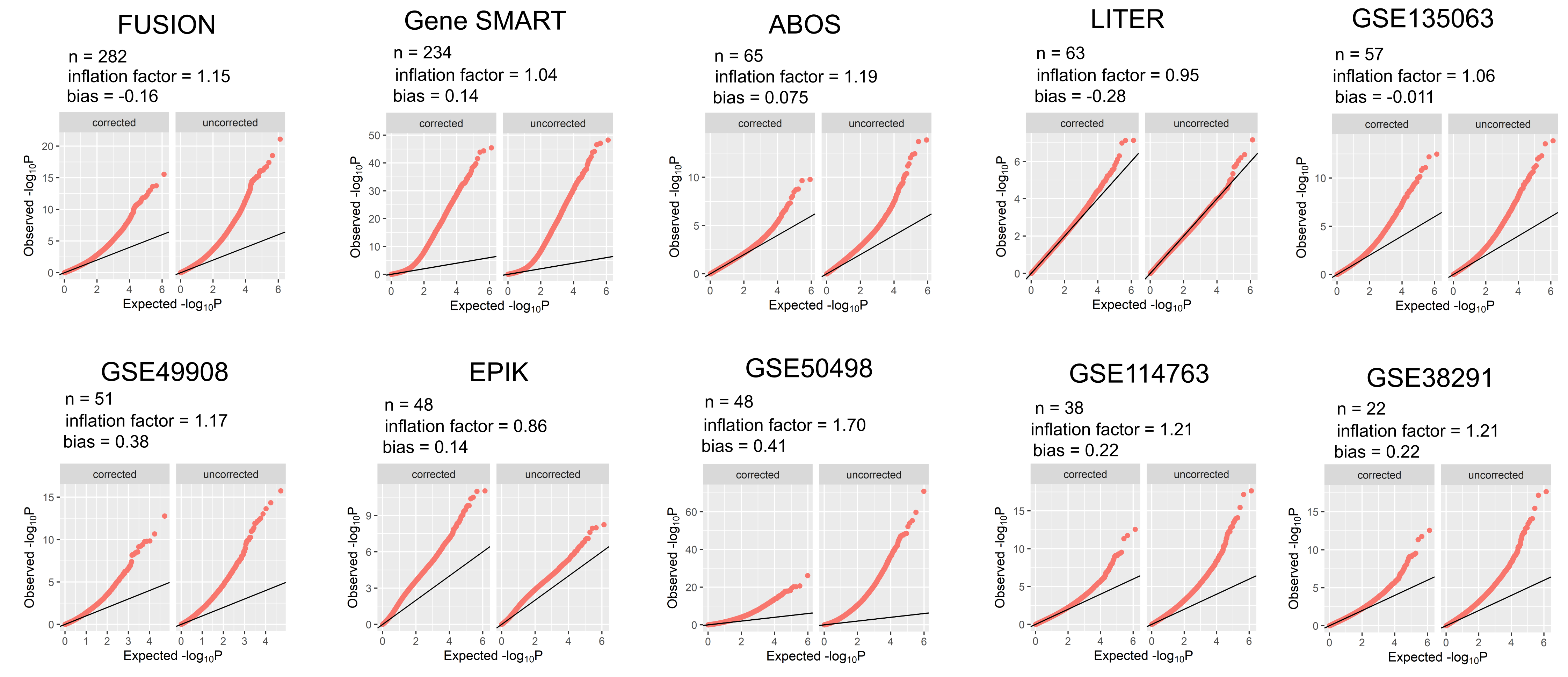

Supplement: Supplementary file 2 — Figure S2. Quantile‐quantile plot of −log10 transformed P‐values for each of the 10 datasets included in the EWAS meta‐analysis. Right panel using uncorrected P‐values and left panel using bacon bias‐ and inflation‐corrected P‐values. [file JCSM-12-1064-s002.png]

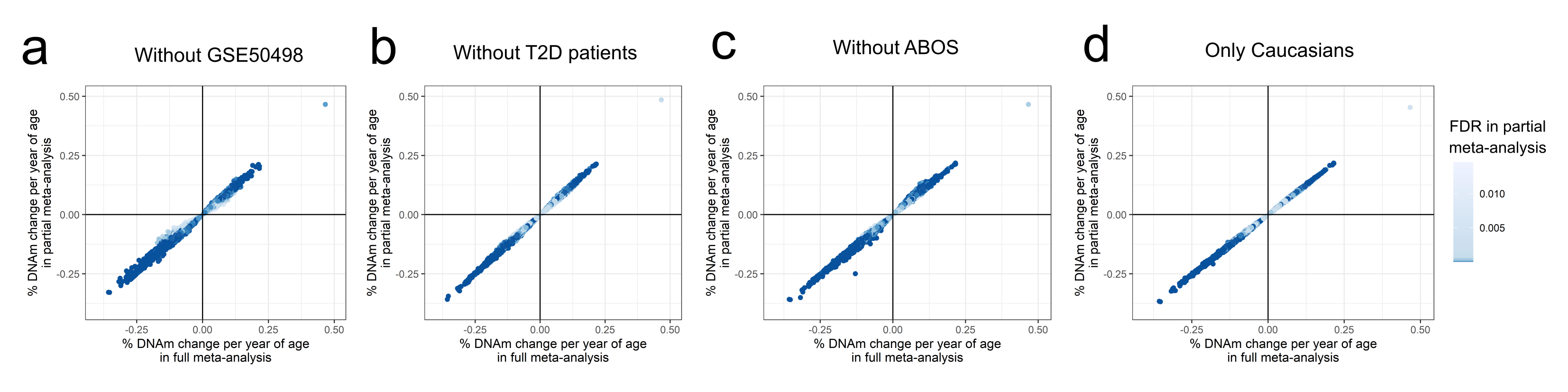

Supplement: Supplementary file 3 — Figure S3. Comparison of results from the full meta‐analysis and from a meta‐analysis excluding GSE50498 (a), type 2 diabetes (T2D) patients (b), the ABOS cohort (c), or non‐Caucasian individuals (d). Each point is one of the 40,479 differentially methylated positions (DMPs) discovered in the full meta‐analysis. To compare results from the full and partial meta‐analyses, we plotted the effect size in the full meta‐analysis (x‐axis), against the effect size in the partial meta‐analysis (y‐axis). To show whether DMPs remained significant in the partial meta‐analysis, we coloured points according to the false discovery rate (FDR) in the partial meta‐analysis. [file JCSM-12-1064-s001.png]
